# Effects of a Reactive Phosphorus–Sulfur Containing Flame-Retardant Monomer on the Flame Retardancy and Thermal and Mechanical Properties of Unsaturated Polyester Resin

**DOI:** 10.3390/polym12071441

**Published:** 2020-06-27

**Authors:** Kang Dai, Zhenzhen Deng, Guyue Liu, Yutong Wu, Wenbin Xu, Yuan Hu

**Affiliations:** 1 School of Environmental Science and Engineering, Guangdong University of Technology, Guangzhou 510006, China; dzz_dengzhenzhen@163.com (Z.D.); liuguyuemail@163.com (G.L.); yutongboom@163.com (Y.W.); xuwenbin@gdut.edu.cn (W.X.); 2 State Key Laboratory of Fire Science, University of Science and Technology of China, Hefei 230026, China

**Keywords:** reactive flame-retardant monomer, unsaturated polyester resin, thermal degradation

## Abstract

A novel reactive phosphorus and sulfur-containing monomer (bis(acryloxyethyldiphenylphosphate)sulfone, BADPS) was synthesized to enhance the comprehensive performance of unsaturated polyester resin (UPR), and corresponding flame-retardant unsaturated polyester resins (FR-UPRs) with various amounts of BADPS were prepared by radical bulk polymerization. The flame retardancy and thermal and mechanical properties of the UPR samples were investigated by limiting oxygen index (LOI) measurements, cone calorimetry, differential scanning calorimetry (DSC), a thermogravimetric analysis (TGA), and a tension test. The results showed that the introduction of BADPS remarkably enhanced the flame resistance and high-temperature stability, as well as the tensile performance of UPR. Scanning electron microscopy (SEM), Fourier transform infrared (FTIR), and Raman spectroscopy studies revealed that BADPS can efficaciously promote the formation of UPR char residue with an improved microstructure and increased graphitization degree, which enhancedthe high-temperature stability and char yield of UPR. Additionally, a thermogravimetry-Fourier transform infrared (TG-FTIR) analysis corroborated that the evolution of combustible volatiles from UPR decomposition was substantially restrained by the incorporation of BADPS, which is beneficial for the suppression of fire hazards in UPR.

## 1. Introduction

As extensively utilized thermosetting materials, unsaturated polyester resins (UPRs) are distinguished by their remarkable mechanical properties, facile processability, excellent chemical resistance, and highly competitive cost. While UPRs have been utilized as matrices in fiber-reinforced composites or alone in industrial applications for several decades, they suffer from the fire risk issue. This issue is mainly accounted for by the high styrene content (ca. 35~40%) and must be overcome to reach the UPRs’ full potential. Thus, the challenge of reducing UPRs’ high flammability has drawn great attention in both industrial and academic fields. Although the desired flame retardancy was achieved by the addition of some halogenated compounds (e.g., tris(2,3-dibromoisopropyl) isocyanurate (TBC) and decabromodiphenyloxide (DBDPO)), it can give rise to severe environmental pollution during combustion and has been forbidden in many fields [1,2]. For many inorganic fillers (e.g., alumina trihydrate and expandable graphite), the high loading of additives and the unfavorable matrix-additive interfacial relationship usually lead to dramatic reductions in the physical and mechanical properties of polymer materials [3,4,5]. As a consequence, these trade-offs should be taken into adequate account in both academic research and industrial applications.

Among many substitutes which meet this challenge in UPRs, reactive flame retardants are prominent candidates because of their characteristics of a low required loading, minimal compromise of the polymers’ intrinsic properties, and tailoring the performance of the materials via the molecular design [6,7]. It is worth noting that, in addition to the environmental friendliness, reactive phosphorus-containing flame retardants have demonstrated convincing flame resistance when bound to polymers, especially those with a high oxygen content, such as polyester and epoxy resin [8,9,10]. Upon the conversion of phosphorus in thermal decomposition, the resulting phosphoric and polyphosphoric species can promote the formation of a cross-linked char layer mainly in the condensed phase [11]. As a stable physical insulator, this char layer can hamper the thermal conductivity and mass transfer, suppressing the combustion of the matrix. Consequently, employing reactive approaches—in terms of incorporating phosphorus-containing moieties into polystyrene, poly (methyl methacrylate), or polyurethane—has been reported by other researchers to significantly enhance the fire retardancy [12,13,14]. Likewise, by introducing phosphorus-containing units such as 9,10-Dihydro-9-oxa-10-phosphaphenanthrene-10-oxide (DOPO) and 9,10-dihydro-10[2,3-di(hydroxycarbonyl)propyl]-10-phosphaphenanthrene-10-oxide (DDP) into UPR, the flame resistance has been greatly improved, both with regard to the high limiting oxygen index (LOI) and low peak heat release rate (PHRR) [15,16,17]. Of particular interest is that most sulfone-containing polymers are endowed with outstanding thermal stability and mechanical properties. Thus, they have been employed to meet the demands of harsh conditions. For example, poly (arylene ether sulphone)s can be manufactured as fire-fighting helmets, and bisphenol S can be substituted for bisphenol A in the polymerization of polycarbonate, resulting in an enhanced thermal tolerance. Notably, the phosphorus element was incorporated into sulfone-containing polymers for improved performance in terms of both flame resistance and thermal properties [6,18,19].Hence, these modified materials can be further used as flame-retardant additives [20,21]. Although phosphorus-containing and sulfone-containing polymers have demonstrated desirable properties in application, few studies have been conducted on the combination of both phosphorus and sulfur elements in a single reactive monomer for polymers prone to fire risk. It should be noted that for phosphate-containing monomers, they may have a negative impact on the glass transition temperature (*T_g_*) as well as the mechanical properties when incorporated into polymers, and this issue can be solved by integrating the sulfone group into a phosphate-containing monomer, as reported in our previous study [22,23,24].

In this work, in an attempt to further explore the applicability of the phosphorus-sulfur combined action on polymers, we synthesized a reactive flame-retardant monomer containing phosphorus and sulfur and then incorporated it into resin to prepare flame-retardant unsaturated polyester resins (FR-UPRs). The flame retardancy and thermal and mechanical properties of the resulting UPR samples were investigated. Moreover, the effects of BADPS on the char formation and thermal decomposition of UPR were discussed, shedding light on the flame-retardant mechanism of FR-UPR.

## 2. Experimental Section

### 2.1. Materials

Hydroxyethyl acrylate (HEA), triethylamine (TEA), tetrahydrofuran (THF), and Bisphenol S were purchased from Sinopharm Chemical Reagent Co. Ltd. (Shanghai, China). Phenyl dichlorophosphate (PDCP) was supplied by Deheng Chemical Corp (Shijiazhuang, China) and was distilled before further use. Benzoyl peroxide (BPO) was recrystallized from methanol for purification. Unsaturated polyester (commercial name 196), containing 38 wt %styrene, was provided by Hefei Chaoyu Chemical Co.Ltd (Hefei, China).

### 2.2. Synthesis and Sample Preparation

#### 2.2.1. Synthesis of Bis (acryloxyethyl diphenyl phosphate) Sulfone (BADPS)

In an ice bath machine, PDCP (21.098 g, 0.1 mol) and THF (100 mL) were firstly fed into a three-neck flask equipped with a dropping funnel, a nitrogen inlet, and a mechanical stirrer. The mixture was stirred vigorously and cooled to −3–0 °C under dry N_2_. Then a mixture of TEA (21.250 g, 0.21 mol) and THF (20 mL) was added dropwise. After 10 min, a solution of HEA (11.612 g, 0.1 mol) in THF (50 mL) was added dropwise for 2 h and maintained at this temperature for an additional 4 h. Subsequently, CuCl (0.030 g) and a solution of Bisphenol S (12.514 g, 0.05 mol) in THF (30 mL) were added into the reaction mixture and heated to reflux for 4 h. After being cooled to room temperature, the mixture was filtered and then concentrated by rotary evaporation. The residue was washed with distilled water, extracted with chloroform, and dried over MgSO_4_, yielding an amber viscous liquid (31.9 g, 84% yield). ^1^H NMR (CDCl3), δ (ppm): 7.15–7.95 (ArH), 6.33–6.42 (1H, CH=CH_2_, cis), 5.96–6.08 (1H, CH=CH_2_), 5.79–5.85 (1H, CH=CH_2_, trans), 4.34–4.52 (4H, O–CH_2_–CH_2_–O); ^31^P NMR (CDCl_3_), δ (ppm): −12.7 (singlet peak). FTIR (KBr pellet, cm^−1^): 1294 (P=O), 1194, 960 (P–O–Ar), 1153 (O=S=O), 1075 (C-S). Element content: C 53.8, O 29.5, H 4.3, P 8.2, and S 4.2 wt %.

#### 2.2.2. Sample Preparation

In different ratios, BADPS was well mixed with unsaturated polyester to prepare the samples, and BPO (2 wt %) was dissolved as an initiator in the mixtures by vigorous mechanical stirring for 0.5 h at room temperature. The resulting homogenized mixtures were placed in an ultrasonic bath (VGT-2013QT, Ningbo Haishudasheng Instrument, Ningbo, China) for 10 min to remove the bubbles generated by stirring. Subsequently, all the mixtures were cast into polytetrafluoroethylene molds, cured at 70 °C for 2 h, and post-cured at 120 °C for 1 h. Finally, transparent yellow solid FR-UPR samples were obtained. According to the concentration of BADPS in the resin, the cured samples are referred to hereafter as BADPS0, BADPS10 (BADPS 10 wt %), BADPS15 (BADPS 15 wt %), and BADPS20 (BADPS 20 wt %). The preparation of the pure BADPS sample followed the same procedures in the UPR. 

### 2.3. Characterization

^1^H NMR and ^31^P NMR spectra were obtained on an NMR spectrometer (AVANCE 300 Bruker, ETH Zurich, Switzerland), and the spectra were referenced by solvent shifts (CDCl_3_ = 7.25 ppm). Fourier Transform Infrared (FTIR) measurements were performed on a FTIR spectrometer (Nicolet 6700, Waltham, MA, USA). A tension test was conducted at a temperature of 23 ± 2 °C using an Instron universal testing machine with a crosshead speed of 0.5 mm/min. At least five samples were tested for each specimen, and the results were averaged in accordance with the standard ASTM D638. Differential Scanning Calorimetry (DSC) was performed on a DSC (Mettler-Toledo, Greifensee, Switzerland) using a heating rate of 20 °C/min from 30 to 250 °C, and the glass transition temperature (*T_g_*) was taken at the inflection point of the curve. A Thermogravimetric Analysis (TGA) was carried out on a IR thermogravimetric analyzer (Q5000, TA Instruments/Waters, Wakefield, MA, USA) under N_2_ or air atmosphere, using a heating rate of 20 °C/min from room temperature to 800 °C in a 60 cm^3^/min stream. All the samples were maintained within 3–5 mg. Limiting Oxygen Index (LOI) measurements were conducted on an HC-2 oxygen index meter (Jiangning Analysis Instrument Co., Nanjing, China) with sample dimensions of 10 × 6.7 × 3 mm^3^, in accordance with standard ASTM D2863-2010. A cone calorimetry test was performed on a cone calorimeter (Fire Testing Technology, West Sussex, UK) in accordance with the standard method ISO 5660, and each specimen (100 × 100 × 3 mm^3^) was wrapped in aluminum foil and exposed horizontally to a 35 kW/m^2^ external heat flux. The morphology of the char residues was studied by a scanning electron microscope (SEM) (Hitachi X650, Hitachi, Tokyo, Japan). Raman spectroscopy measurements were conducted at room temperature with a laser Raman spectrometer (SPEX-1403, Metuchen, New Jersey, USA), and the Gaussian peak type in the Peak Fitting Module of Origin 8.0 software was employed in curve fitting to determine the spectral parameters. Thermogravimetry-Fourier transform infrared (TG-FTIR) spectroscopy was performed using a IR thermogravimetric analyzer (Q5000, TA Instruments/Waters, Wakefield, MA, USA) interfacing with a FTIR spectrometer (Nicolet 6700, Madison, WI, USA) under an N_2_ atmosphere. 

## 3. Results and Discussion

### 3.1. Structural Characterization of BADPS

**Scheme 1:**
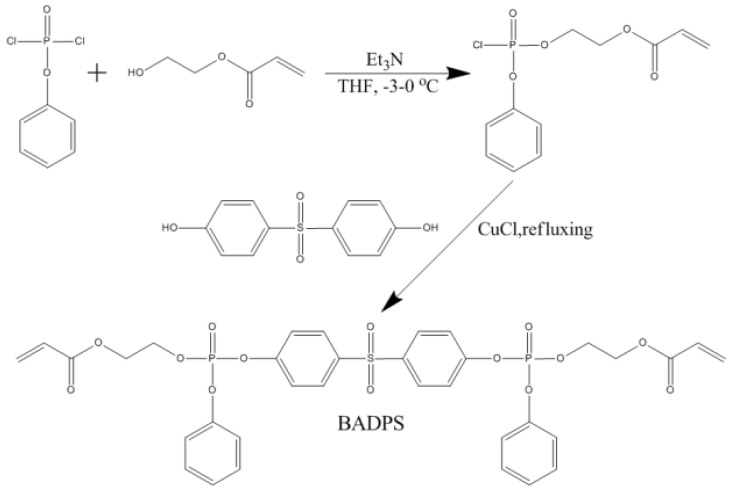
Synthetic pathway of bis(acryloxyethyldiphenyl phosphate)sulfone(BADPS).

**Figure 1:**
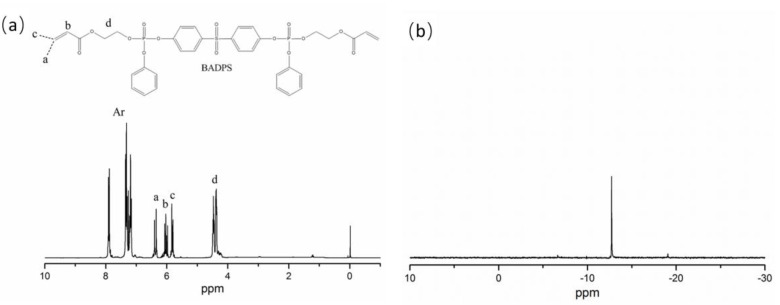
(**a**) ^1^H NMR and (**b**) ^31^P NMR spectra of BADPS.

As illustrated in Scheme 1, the synthetic procedures of BADPS are first the reaction of PDCP with HEA, followed by esterification with Bisphenol S, and the structural characterization of BADPS in the ^1^H NMR and ^31^P NMR spectra are shown in Figure 1. In the ^1^H NMR spectrum, the chemical shifts δ = 7.15–7.95 ppm are attributed to aromatic protons and, specifically, the four aromatic protons close to sulfone group are responsible for the shifts δ = 7.83–7.95 ppm [25]. Moreover, the vinylic signals can be observed at 5.79–6.42 ppm, and the signals appearing at 4.34–4.52 ppm correspond to the methoxy protons [22,25]. Additionally, in the ^31^P NMR spectrum a single peak at −12.7 ppm further corroborates the high purity of BADPS. 

**Figure 2:**
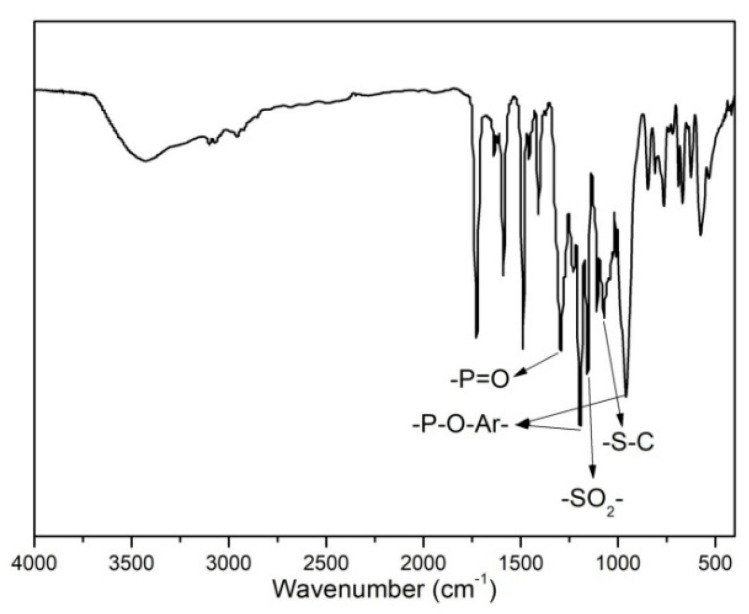
FTIR spectrum of BADPS.

With respect to the FTIR spectrum of BADPS in Figure 2, two obvious peaks appear at 960 and 1194 cm⁻^1^, corresponding to the vibration of P–O–Ar [26]. The absorption at 1294 cm⁻^1^ is characteristic of P=O. Meanwhile, the symmetric stretching vibration of sulfone (1153 cm⁻^1^) and the stretching vibration of aromatic C-S (1075 cm⁻^1^) can be clearly observed [27]. All of these results conform to the expected structure of BADPS.

### 3.2. Mechanical Properties 

The impact of BADPS on the mechanical properties of UPR (i.e., tensile strength, Young’s modulus, and strain to failure) was evaluated by a tension test. Interestingly, with a 10 wt % BADPS content, the UPR sample showed a substantial improvement in tensile performance, as presented in Table 1. Concurrent with a ~260% increase in the elongation at break, the tensile strength increased from 33.62 to 47.49 MPa, affording BADPS10 a toughness ~446% higher than that of BADPS0 (toughness is defined here as the area under the stress–strain curve) [28]. Simultaneously, BADPS10 still maintained a Young’s modulus of 2737 MPa, which is somewhat lower than the 3062 MPa for BADPS0. Although the further incorporation of BADPS enhanced the ductility of the UPR samples, as the elongation at break for BADPS15 and BADPS20 increased to 3.57% and 3.85%, respectively, the tensile strength and toughness declined, and the Young’s modulus was reduced by 23% when the BADPS content reached 20 wt %. These results can be attributed to the alterations in the chemical composition of UPRs after the incorporation of BADPS–aryl–ether linkage can enhance toughness, and both aryl and sulfone groups contribute to adequate chain rigidity and polarity. All of these factors are critical for improved tensile strength [29]. When the BADPS content rose above 15 wt %, numerous alkyl and ether groups facilitated the free rotation of polymer links to a great extent [30], and the resins exhibited much improved ductile properties. As a consequence, the elongation at break further increased, whereas the tensile strength and toughness began to decrease from the maximums at 10 wt % BADPS, and the increasing ductility can account for the decrease in the Young’s modulus. Nonetheless, the enhancements in the mechanical properties are still evident, as the toughness achieved a fourfold increase in the presence of 20 wt % BADPS. In many cases, the application of thermosetting resins, such as epoxies and UPRs, in composites is limited by their brittle nature, and thus the reported properties will be particularly desirable in these situations.

### 3.3. Thermal Properties

**Figure 3:**
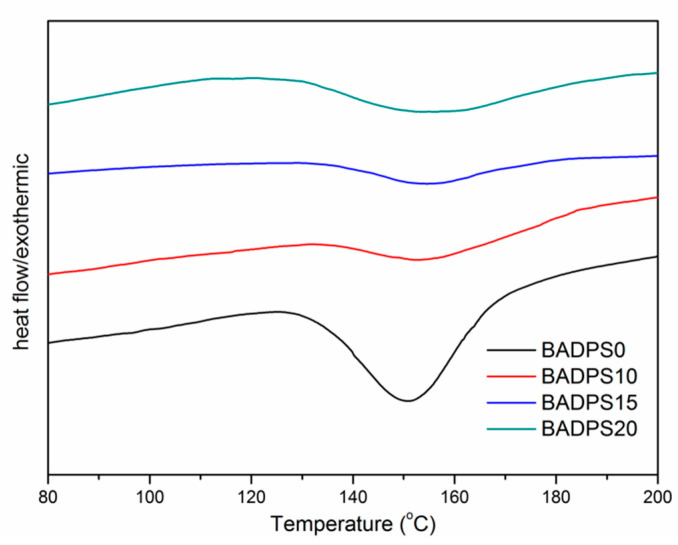
Differential scanning calorimetry (DSC) curves of the different UPRs.

From an industrial application perspective, the operating temperature range of a material principally depends on its *T*_g_. Explicitly, the higher the *T*_g_ of a polymer material, the wider the operating temperature range is. Further, a combination of factors, such as cross-linking degree, chain rigidity, and polarity, greatly influence the glass transition of the polymer [30,31]. In the case of BADPS, when introduced into UPR the two double bonds structure increased the UPR’s cross-linking degree and, coupled with the presence of high polar sulfone links and abundant rigid aryls, led to a marked enhancement in *T*_g_ for the UPR. As confirmed by DSC (Figure 3), a higher *T*_g_ was observed for UPRs containing BADPS as opposed to BADPS0. Remarkably, the *T*_g_ value increased from 150.6 to 155.8 °C when the amount of BADPS reached 20 wt %.

**Figure 4:**
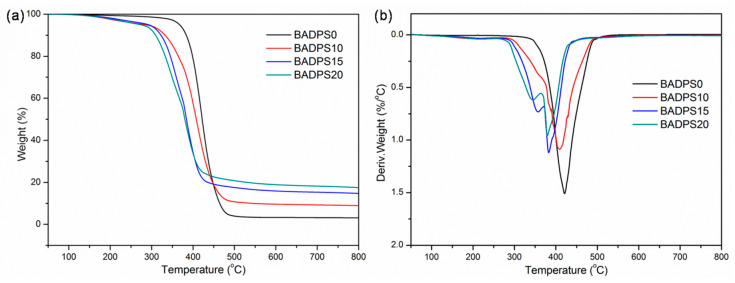
(**a**) TG and (**b**) DTG curves of the different UPRs in an N_2_ atmosphere.

**Figure 5:**
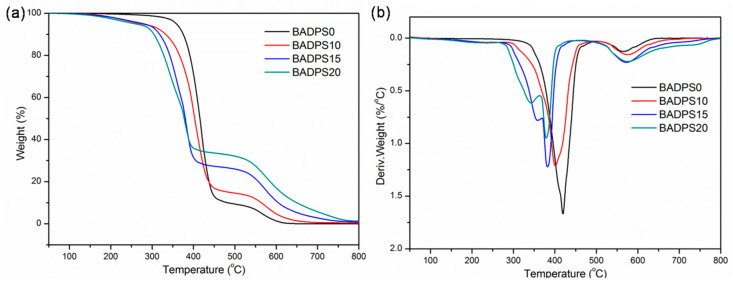
(**a**) TG and (**b**) DTG curves of the different UPRs in air.

As the results of the thermal analysis demonstrated, the introduction of BADPS into the UPR substantially altered the decomposition process. Under nitrogen, all of the UPRs containing BADPS underwent two-stage decomposition, whereas a single stage process was observed for the neat UPR, as shown in Figure 4. In the case of the samples incorporating BADPS, the broad early mass loss step corresponds to the cleavage of the relatively weak P–O–C linkage in BADPS, and the temperature at 10% weight loss (*T*_0.1_) decreased by 74 °C in a 20 wt% BADPS content in comparison with that of BADPS0 [18]. Additionally, the maximum mass loss rate (MMLR), as obtained from the DTG data, was significantly lowered by the incorporation of BADPS. Specifically, the MMLRs for BADPS15 and BADPS20 were reduced by 26% and 37%, respectively, relative to that of the neat UPR. The great improvement in char formation is also worthy of note. After the dehydration and chain scissoring in the principal weight loss (250–500 °C) [11], only a 3.3 wt % char yield was observed for BADPS0 at 700 °C, whereas the char yield of BADPS20 was as high as 18.2 wt%, indicating that the resulting char is stable in this inert atmosphere. In air, as shown in Figure 5 and Table 2, in addition to another stage of weight loss for all the UPR samples, the *T*_0.1_ was somewhat lower than that under nitrogen. These alterations imply that the polymer degradation may be accelerated by oxygen. Moreover, the presence of oxygen can further the degradation of the primary carbonaceous char, as observed after ca. 470 °C in Figure 5. Remarkably, there was no char residue for BADPS0 at 700 °C. However, BADPS20 presented a 5.7 wt % char yield, demonstrating a significant enhancement in the thermo-oxidative stability. With respect to the MMLR, a 43% reduction was observed in 20 wt % BADPS relative to that of BADPS0.

**Figure 6:**
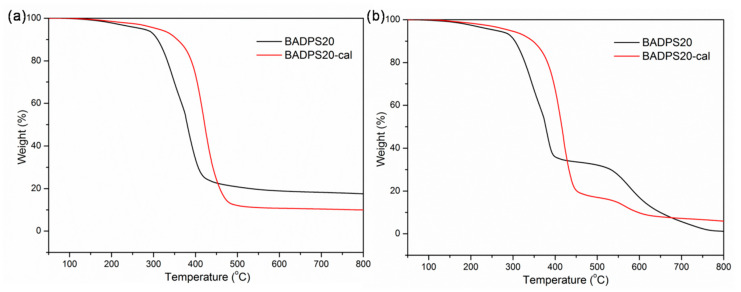
Experimental and theoretical TG curves of BADPS20 in (**a**) N_2_ atmosphere and (**b**) air.

Since in both nitrogen and air atmospheres the char formation as well as the high-temperature stability of UPR char was substantially enhanced by the incorporation of BADPS, further investigation of the interactions between the UPR and BADPS was conducted by examining the weight difference between the experimental and theoretical TG curves of BADPS20, as illustrated in Figure 6. The theoretical TG curve was calculated by a linear combination of the experimental TG curves of the neat UPR and BADPS (a cured sample) according to their percentages. In nitrogen, the initial degradation of BADPS20 occurred earlier than that of BADPS20-cal, as the *T*_0.1_ for BADPS20 was reduced by 47 °C. Moreover, above 500 °C, the experimental char yield of BADPS20 was much higher than the calculated value. These differences suggest that BADPS was chemically reacting with UPR during degradation and can promote char formation through cross-linking reactions between phosphate ester groups and UPR molecular chains [32]. Interestingly, at 500 °C the difference between the experimental and theoretical char yields of BADPS20 in air (Figure 6b) is greater than that under nitrogen atmosphere (Figure 6a). This difference in char yield can be explained by the facilitation of char formation by the oxygen in air. In most cases of the condensed phase flame retardant action of phosphorus among polymers, phosphorus promotes char formation in the form of dehydrating phosphoric acid [33]. The higher the oxidation state of phosphorus, the stronger the acid will be, with an improved ability to promote char production [34]. In air, the presence of oxygen might have a positive effect on the formation of phosphorus-based acids, leading to enhanced performance in charring. Additionally, the sulfone group in BADPS can accelerate the cross-linking reaction for char formation [35]. These combined effects facilitated the promoted char yield in UPR. The resulting primary BADPS20 carbonaceous char, however, was further oxidized at higher temperatures, and the corresponding char yield above 680 °C was lower than that of BADPS20-cal. Considering that no neat resin char residue remained at these temperatures, the char yield of BADPS20-cal can be accounted for by the existence of BADPS char, owing to its much better thermo-oxidative stability.

From these results, it may be concluded that BADPS with a characteristic molecular structure can contribute a higher *T*_g_ to UPR_._ Furthermore, the UPR decomposition process was significantly altered by the introduction of BADPS in both the air and nitrogen atmospheres, enhancing the thermal behavior and char yield of UPR in high-temperature regions. Thus, the resulting char can serve as an efficient insulator, protecting the substrate from further degradation.

### 3.4. Flame Retardancy

The flame retardancy of the UPR was remarkably enhanced by the incorporation of BADPS, as measured in the LOI test. Although the pristine UPR (BADPS0) is highly flammable, with an LOI value of 20.5, the LOI value reached 24.5 for BADPS10 and further increased to 26.5 for BADPS20, as shown in Table 3. Meanwhile, it can be noted that while fire dripping was seen for the neat UPR, after incorporation of BADPS no dripping occurred in the FR-UPRs. This can be attributed to the much enhanced charring performance in FR-UPRs, as demonstrated by the TGA results.

**Figure 7:**
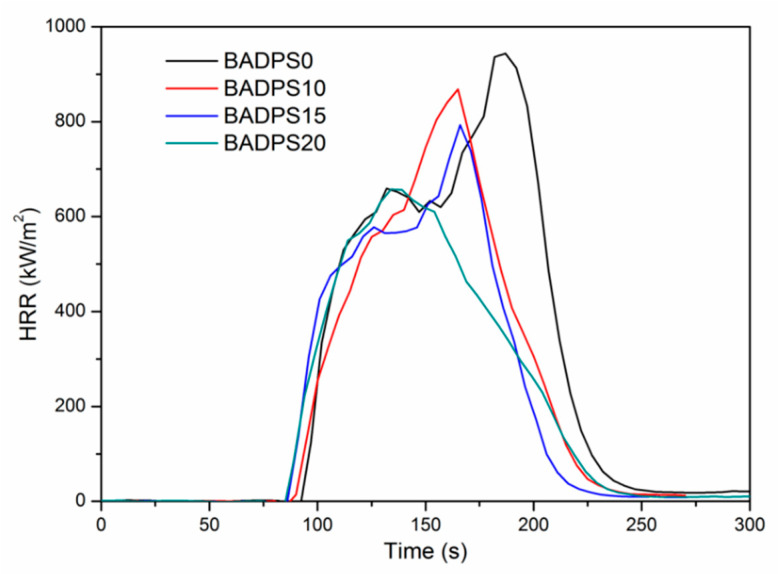
Heat release rate (HRR) curves of the different UPRs.

**Figure 8:**
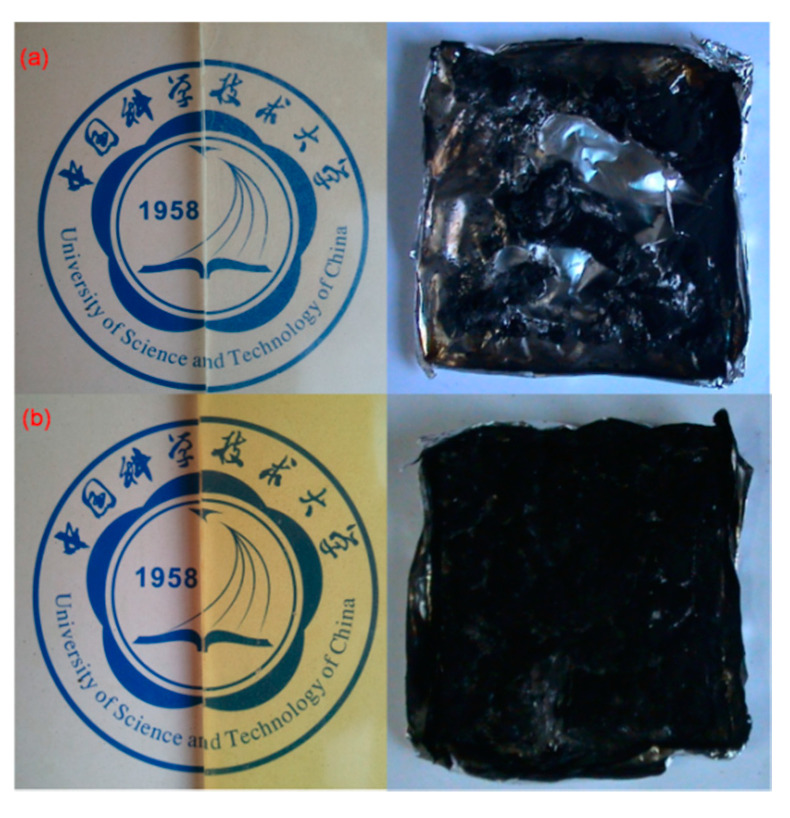
Photo images of samples before and after the cone calorimetry test: (**a**) BADPS0, (**b**) BADPS20.

Cone calorimetry fits reasonably well with the results in large-scale fire tests and thus can satisfactorily describe the fire behavior of materials [36]. Notably, many parameters (e.g., heat release rate (HRR), peak heat release rate (PHRR), time to ignition (TTI), total heat release (THR), and effective heat of combustion (EHC)) can be obtained from cone measurements for comprehensive evaluation [37,38]. The HRR curves of the UPRs and corresponding cone calorimetry data are presented in Figure 7 and Table 3, respectively. Compared to the high PHRR value of the neat UPR, a significant reduction in the PHRR value was observed for UPRs containing BADPS, decreasing from 944 kW/m^2^ (BADPS0) to 657 kW/m^2^ (BADPS20). It is worth noting that the mass residue after burning increased as the proportion of BADPS in the UPRs rose (see Figure 8), suggesting the occurrence of the carbonization of more UPR molecular chains during pyrolysis [39]. Thus, the resulting more cohesive char can act as a robust insulator to protect the polymer from thermal degradation. Meanwhile, as more char was left behind, the THR decreased. As a consequence, the THR value of the polymer incorporating BADPS was observed to be much lower than that of the pure UPR, with a 17.5% and 27.5% reduction for BADPS10 and BADPS20, respectively, as can be seen in Table 3. To summarize, the performance of UPRs in terms of the LOI and cone tests suggests that the introduction of BADPS into UPR can greatly reduce the flammability of UPR.

### 3.5. Char Residue Morphological Study and Spectroscopic and Element Analyses

**Figure 9:**
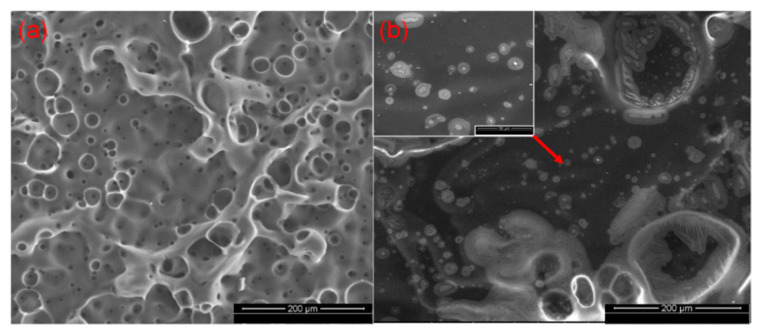
SEM images of the char residues for (**a**) BADPS0 and (**b**) BADPS20.

To further our understanding of the flame retardant mechanism of FR-UPRs, after the cone calorimetry test the resulting residual char was investigated by SEM. In the case of BADPS0, the char surface appears to be porous with numerous pits (see Figure 9a). However, an entirely different surface morphology can be observed for the BADPS20 char. Apart from the non-porous and less pitted surface (even at a higher magnification), the BADPS20 char (see Figure 9b) shows some robust bubbles, which are beneficial for the protection of the substrate from thermal feedback.

**Figure 10:**
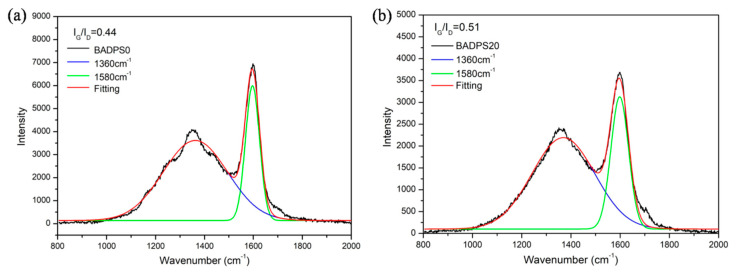
Raman spectra of the char residues for (**a**) BADPS0 and (**b**) BADPS20.

In recent years, since Raman spectroscopy is sensitive to both crystal structures and molecular structures, it has been employed for the characterization of carbonaceous materials. The char microstructures of BADPS0 and BADPS20 were also investigated by Raman spectroscopy in the present paper. As shown in Figure 10, the spectra of both samples exhibit two highly overlapping diffusion bands. The former, at ca.1580 cm⁻^1^ (G band), is assignable to an ideal graphitic lattice stretching vibrational mode with E_2g_ symmetry, whereas the latter, at ca.1360 cm⁻^1^ (D band), accounts for the graphite structural disorder [40]. Specifically, the ratio of the integrated G and D bands intensities (*I*_G_/*I*_D_) by peak fitting can present the degree of graphitization in materials (i.e., the higher the value of *I*_G_/*I*_D_, the better the microstructure of the char is and with fewer defects). In comparison with the 0.44 *I*_G_/*I*_D_ value of pure UPR, BADPS20 obtained a higher *I*_G_/*I*_D_ value (0.51), revealing that the UPR char microstructure was considerably improved by the incorporation of BADPS.

**Figure 11:**
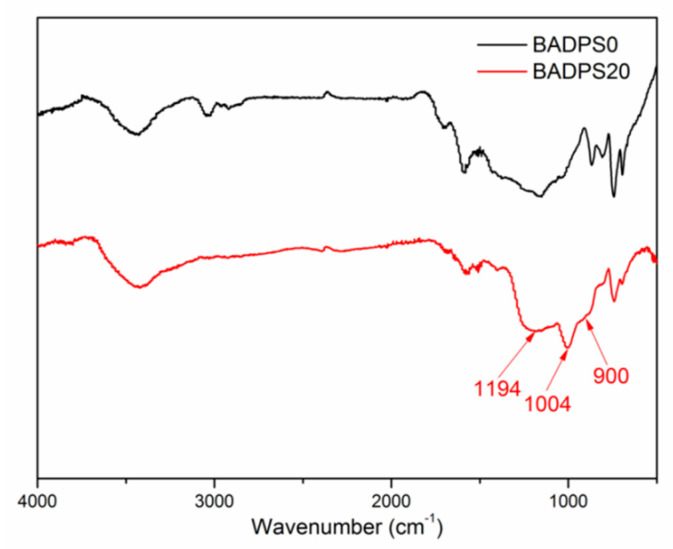
FTIR spectra of char residues for BADPS0 and BADPS20.

The FTIR spectra of the char residues are shown in Figure 11, and the main variations in the characteristic absorption bands between 1400 and 900 cm⁻^1^ are illustrated. In particular, for BADPS20 the evident sharp absorption peak at 1004 cm⁻^1^ is ascribed to the symmetrical stretching of PO_2_ and PO_3_ in phosphocarbonaceous complexes [41]. The broad peak at 1194 cm⁻^1^, characteristic of P–O–Ar, further corroborates the formation of a phosphocarbonaceous structure [26]. Moreover, another broad peak, corresponding to the asymmetric stretching vibration of P–O–P in polyphosphate species, was observed at 900 cm⁻^1^, and this suggests that the phosphate species were cross-linked to form a more complex structure [34]. Consequently, char residue with an improved cross-linked aromatic microstructure can act as an effective barrier to impede mass transfer and thermal diffusion. It should be noted that no clear absorption band indicative of sulfone group was found in the spectrum of the BADPS20 char residue. In addition, an element analysis of the char residues (see Table 4) further gives evidence that most of the phosphorus was left in the char residue, whereas very limited sulfur was maintained. Studies have shown that in sulfone-containing polymers, the Ar–SO_2_ bond undergoes thermal cleavage during combustion, and then most of the resulting fragments are released in the form of sulfur dioxide and sulfone-containing aromatic compounds [6,42,43]. These volatiles, according to the previous report, can facilitate the generation of CO_2_, dilute combustion products, and promote char formation [35]. Hence, the combination of phosphate and sulfone in BADPS effectively improved the fire behavior of UPR.

### 3.6. TG-FTIR Analysis of the Volatiles

**Figure 12:**
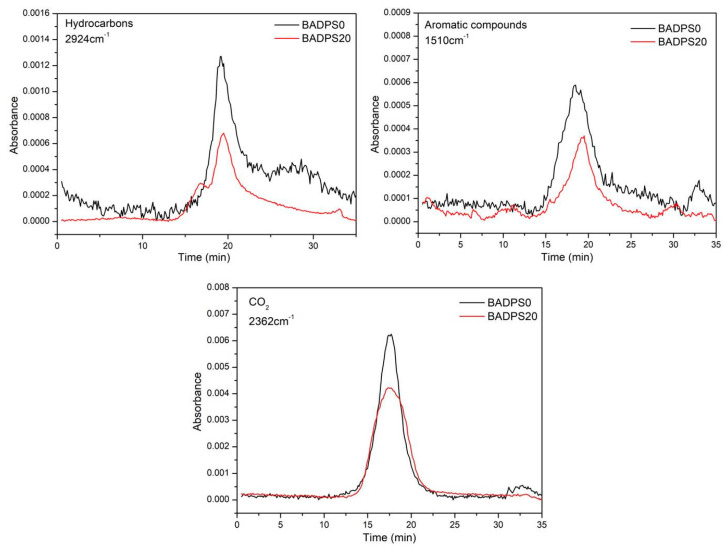
TG-FTIR spectra of pyrolysis products for BADPS0 and BADPS20.

To gain insight into the influence of BADPS on the evolved volatiles of UPR during pyrolysis, TG-FTIR spectroscopy was employed to analyze the volatile components released from BADPS0 and BADPS20. The absorbance-time curves of the selected representative pyrolysis products, including hydrocarbons, aromatic compounds, and carbon dioxide, are exhibited in Figure 12, and the absorbance values were normalized. It can be seen that the absorbance intensity of hydrocarbons and aromatic compounds in BADPS20 is notably lower than that in BADPS0. Since these organic volatiles act as “fuel” during combustion, the reduction in the amount of combustible products can account for the decreased HRR, THR, and EHC in UPR, in good accordance with the cone calorimetry data. Meanwhile, it can be noted that the intensity of the nonflammable gas carbon dioxide is decreased in BADPS20, corresponding to the improved char formation in TGA results. E. Kandare et al. reported that the mainly gaseous products from the pyrolysis of UPR are carbon dioxide, anhydrides, and aromatic species [11]. The significant reduction in the absorbance intensity of the relative pyrolysis products, as demonstrated in Figure 12, gives evidence that the introduction of BADPS can inhibit the release of combustible gas.

## 4. Conclusions

In this work, a novel reactive phosphorus-and sulfur-containing monomer (BADPS) was synthesized and well characterized by ^1^H NMR, ^31^P NMR, and FTIR. The monomer with different amounts was incorporated into unsaturated polyester by radical bulk polymerization. The resulting FR-UPRs exhibited a higher *T_g_*, enhanced tensile strength, and better toughness as compared to those of the pure UPR. These improvements in UPR are attributed to the characteristic molecular structure of BADPS. Furthermore, the TGA results suggested that the introduction of BADPS notably improved the high-temperature stability of UPR, demonstrating a higher char yield and much reduced MMLR. The flame retardancy of UPR was significantly enhanced after the incorporation of BADPS, with an increased LOI and reduced PHRR and THR. The morphological studies and spectroscopic analyses revealed that the presence of BADPS in UPR effectively promoted char formation during the thermal decomposition and endowed the char residue with a better microstructure and a higher degree of graphitization. The resulting robust char residue can serve as a physical barrier to hinder gaseous volatiles release, reduce heat diffusion, and suppress the combustion of the UPR matrix. In brief, the combination of phosphate and sulfone in BADPS effectively enhanced the integration properties of UPR, providing a foundation for further UPR study.

## Figures and Tables

**Table 1:** Mechanical properties of the different unsaturated polyester resin (UPRs).

Sample	Tensile Strength (MPa)	Young’s Modulus (MPa)	Elongation at Break (%)	Toughness
BADPS0	33.62 ± 3.13	3062 ± 97	1.35 ± 0.17	24.6 ± 2.9
BADPS10	47.49 ± 2.58	2737 ± 89	3.50 ± 0.33	109.7 ± 2.7
BADPS15	45.42 ± 3.10	2657 ± 116	3.57 ± 0.27	106.3 ± 3.2
BADPS20	37.11 ± 2.11	2371 ± 78	3.85 ± 0.21	101.1 ± 2.4

**Table 2:** Glass transition temperature (*T_g_*) and thermogravimetric analysis (TGA) data in air and N_2_ atmospheres for the different UPRs.

Sample	*T_g_* (°C)	*T*_0.1_ (°C)		*T*_max_ (°C)		MMLR (%/°C)		Char (500 °C, wt%)		Char (700 °C, wt%)	
		Air	N_2_	Air	N_2_	Air	N_2_	Air	N_2_	Air	N_2_
BADPS0	150.6	375	383	419/564	421	1.67	1.51	9.4	4.0	0	3.3
BADPS10	152.7	331	333	401/574	409	1.21	1.09	14.6	10.8	0.6	9.3
BADPS15	154.6	318	322	358/382/573	357/383	1.22	1.12	26.0	17.6	2.8	15.3
BADPS20	155.8	304	309	342/379/575	344/380	0.95	0.96	32.1	20.8	5.7	18.2

**Table 3:** Cone calorimetry data for the different UPRs.

Sample	BADPS Content (wt %)	LOI	TTI ^a^ (s)	PHRR (kW/m^2^)	THR (MJ/m^2^)	Mass Residue (%)	Mean EHC ^b^ (MJ/kg)
BADPS0	0	20.5	97	944	80	2.1	19.5
BADPS10	10	24.5	95	869	66	3.7	16.6
BADPS15	15	25.5	91	793	61	8.7	16.6
BADPS20	20	26.5	89	657	58	9.5	16.5

^a^ Time to ignition, ^b^ effective heat of combustion.

**Table 4:** P and S element analysis of the char residues for UPRs.

Element Content	BADPS0	BADPS20
P (%)	0	11.6
S (%)	0	0.5
